# Diagnostic and Prognostic Significance of miR-155, miR-181, miR-221, miR-222, and miR-223 Expression in Myelodysplastic Syndromes and Acute Myeloid Leukemia

**DOI:** 10.3390/diagnostics16010013

**Published:** 2025-12-19

**Authors:** Cemile Ardıç, Mustafa Ertan Ay, Kenan Çevik, Anıl Tombak, Özlem İzci Ay, Ümit Karakaş, Gurbet Doğru Özdemir, Abdulkadir Bilgiç, Mehmet Emin Erdal

**Affiliations:** 1 Department of Medical Biology and Genetics, Faculty of Medicine, Mersin University, Mersin 33343, Türkiye; ardiccemile@gmail.com (C.A.); cvkknn@gmail.com (K.Ç.); ozlemay@mersin.edu.tr (Ö.İ.A.); grbtdgr@gmail.com (G.D.Ö.); mehmeteminerdal@gmail.com (M.E.E.); 2 Department of Hematology, Faculty of Medicine, Mersin University, Mersin 33343, Türkiye; aniltombak@mersin.edu.tr; 3 Department of Pharmacy Services, Health Services Vocational School, Bayburt University, Bayburt 69000, Türkiye; umitk.kas@gmail.com; 4 Department of Cardiovascular Surgery, Faculty of Medicine, Mersin University, Mersin 33343, Türkiye; bilgicabdulkadir@gmail.com

**Keywords:** myelodysplastic syndrome, acute myeloid leukemia, miR-155, miR-181, miR-221, miR-222, miR-223, biomarker

## Abstract

**Background:** Myelodysplastic syndromes (MDSs) and acute myeloid leukemia (AML) are clonal hematological disorders that share molecular origins but present with distinct clinical features. MicroRNAs (miRNAs) are key post-transcriptional regulators, and their altered expression may reflect biological shifts contributing to disease progression. **Methods:** Expression levels of miR-155, miR-181, miR-221, miR-222, and miR-223 were analyzed by RT-qPCR in bone marrow samples from 37 MDS patients, 20 AML patients, and 7 controls. Group comparisons were performed using ANOVA (with Benjamini–Hochberg correction) and Tukey post hoc testing. Diagnostic performance and network behavior were evaluated using ROC analysis, Pearson correlation matrices, and principal component analysis (PCA). **Results:** miR-155, miR-181, and miR-223 were upregulated in AML, whereas miR-221 and miR-222 were downregulated. miR-222 showed the highest diagnostic accuracy (AUC ~0.87 for both AML vs. control and MDS vs. control). Its expression was significantly higher in high IPSS-R MDS cases (*p* = 0.046), with a similar upward tendency for miR-221 (*p* = 0.054). Progressive loss of coordinated miRNA expression was observed from controls to MDS and AML. PCA supported these findings by showing separation mainly driven by miR-222 and miR-155. **Conclusions:** Combined miRNA profiling highlights miR-222 and, to a lesser extent miR-155, as consistent indicators of myeloid disease transformation. While further validation in larger and genetically stratified cohorts is warranted, these findings support the potential contribution of miRNA signatures to diagnostic evaluation and risk stratification in MDS and AML, in line with precision hematology approaches.

## 1. Introduction

Myelodysplastic syndromes (MDSs) and acute myeloid leukemia (AML) are related clonal disorders of hematopoietic stem or progenitor cells characterized by ineffective hematopoiesis, cytopenias, and varying propensities for progression to AML [1]. While recent genomic studies have clarified the mutational and epigenetic landscape of these entities, the intermediate molecular mechanisms linking early dysplasia to overt leukemia remain incompletely understood [1,2].

MicroRNAs (miRNAs) are small non-coding RNAs that fine tune gene expression at the post-transcriptional level by binding to complementary sites on target mRNAs. They regulate proliferation, apoptosis, and differentiation, thereby maintaining hematopoietic balance [3]. When dysregulated, miRNAs contribute to the altered gene expression patterns characteristic of myeloid neoplasms and have been proposed as biomarkers for disease classification, prognosis, and therapeutic response.

Aberrant expression of several miRNAs, including miR-155, miR-181, miR-221, miR-222, and miR-223, has been repeatedly reported in MDS and AML [4,5]. These molecules regulate inflammatory signaling, cell-cycle control, and granulocytic maturation, yet reported expression trends vary among studies, possibly reflecting differences in disease stage, genetic background, or sample type. More recent AML and MDS cohorts have further linked these miRNAs to cytogenetic risk, treatment response, and outcome, underscoring their translational relevance [6,7,8,9].

miR-155 is a well-characterized oncomiR that supports leukemic proliferation and inflammatory signaling [10,11]. High miR-155 expression correlates with poor prognosis in cytogenetically normal AML [12]. The miR-181 family regulates differentiation via BCL-2, HOXA9, and MEIS1 and shows context-dependent roles-upregulated in favorable AML but decreased in high-risk MDS and secondary AML, and in some settings associated with increased chemosensitivity [13,14,15].

The miR-221/222 cluster, located on the X chromosome, regulates key effectors of hematopoietic proliferation and differentiation, including c-KIT, p27^Kip1^, and p57^Kip2^ [16]. In MDS, evidence directly linking miR-221/222 expression to IPSS-R stratification is currently limited. However, similar associations between higher miRNA expression and increased risk categories have been demonstrated, such as miR-196b-5p showing elevated levels in higher-risk MDS and secondary AML cases [17]. In contrast, AML more consistently exhibits reduced miR-221/222 expression, particularly in aggressive subtypes [18,19,20]. Functionally, dysregulation of these miRNAs may influence leukemic progression through oncogenic pathway activation. Notably, miR-222 has been shown to enhance cellular proliferation by targeting Axin2 and activating the WNT/β-catenin pathway [21].

miR-223 promotes granulocytic maturation by regulating E2F1, FBXW7, and MEF2C [22,23,24]. Its loss leads to impaired differentiation and excessive proliferation, typical of AML and advanced MDS [23]. Consistent with this, recent clinical data indicate that circulating miR-223 levels are significantly reduced in AML, particularly in patients with intermediate- or high-risk cytogenetic profiles, and lower serum miR-223 is associated with more aggressive disease and shorter overall survival [25]

Collectively, these miRNAs converge on key hematopoietic regulatory pathways, NF-κB/STAT (miR-155), BCL2-HOXA9-MEIS1 (miR-181), c-KIT/p27/p57 (miR-221/222), and E2F1/FBXW7/MEF2C (miR-223), which coordinate cellular proliferation, differentiation, and survival [10,11,16,22,23,24,26]. However, most previous studies have investigated individual miRNAs or restricted subsets, often focusing on either AML or MDS in isolation and employing heterogeneous methodological approaches. To the best of our knowledge, integrated analyses directly comparing this five-miRNA panel across the MDS-AML continuum and evaluating their combined network behavior in human bone marrow samples remain limited.

This study investigates the expression of miR-155, miR-181, miR-221, miR-222, and miR-223 in bone marrow samples from MDS and AML patients and controls. Using bone marrow as the primary tissue enhances clinical relevance, as it reflects the main site of disease evolution. Our aim is to define diagnostic expression signatures, explore associations with IPSS-R risk categories, and characterize how deregulated miRNA networks relate to disease heterogeneity and progression from MDS to AML.

## 2. Materials and Methods

### 2.1. Study Population

Bone marrow (BM) samples were obtained from 20 newly diagnosed and previously untreated patients with AML (10 males, 10 females; median age 62 years; range 43–77 years), 37 newly diagnosed and previously untreated patients with MDS (20 males, 17 females; median age 69 years; range 50–88 years), and 7 control individuals (5 males, 2 females; median age 64 years, range 55–76 years). The control group consisted of individuals without hematologic disorders, from whom bone marrow fluid was collected by aspiration from the surgical field after the sternum was opened during cardiac surgery. The number of control samples was limited because bone marrow aspiration from healthy individuals is ethically restricted and may only be performed in cases of medical indication or prior diagnostic necessity. MDS cases were classified according to the WHO 2016 criteria, and AML diagnoses were based on FAB classification, bone marrow biopsy findings, peripheral blood counts, and flow cytometry.

All participants provided written informed consent, and the study protocol was approved by the Institutional Ethics Committee (No: 2016/344). The main clinical and hematological characteristics are summarized in Table 1.

### 2.2. RNA Extraction and cDNA Synthesis

Total RNA was isolated from bone marrow cells using TRIzol reagent (Invitrogen, Thermo Fisher Scientific, Waltham, MA, USA). RNA concentration and purity were measured with a NanoDrop 2000 spectrophotometer (Thermo Fisher Scientific, Waltham, MA, USA). To enhance small RNA yield, the TRIzol extraction protocol was optimized by incorporating additional ethanol precipitation and extended isopropanol incubation, in line with standard adaptations used for miRNA isolation.

Complementary DNA (cDNA) was synthesized from 2 µg of total RNA using Revertaid Reverse Transcriptase (Thermo Fisher Scientific, Vilnius, Lithuania) following the manufacturer’s protocol. Reverse transcription was performed at 37 °C for 60 min and 95 °C for 5 min. cDNA samples were stored at −20 °C until analysis.

### 2.3. Quantitative Real-Time PCR (RT-qPCR)

Expression levels of miR-155, miR-181, miR-221, miR-222, and miR-223 were measured using the ABI Prism 7500 Real-Time PCR System (Applied Biosystems, Foster City, CA, USA) and ZNA™ fluorogenic probes (Metabion International AG, Planegg/Steinkirchen, Germany). ZNA probes were custom-designed by the research team based on mature miRNA sequences (miRBase v22) and synthesized commercially. Assay specificity and performance were verified via melting curve analysis and amplification efficiency. Primer and probe sequences are listed in Table 2. Each 25 µL reaction contained 12.5 µL of TaqMan Gene Expression Master Mix, 5 µL of cDNA, 2.5 µL of primer mix (900 nM each), 0.4 µL of probe (200 nM), and nuclease-free water. The cycling protocol was 50 °C for 2 min, and 95 °C for 10 min, followed by 50 cycles of 95 °C for 15 s and 60 °C for 1.5 min. All reactions were performed in duplicate. Expression values were normalized to hsa-miR-26b-5p, and relative quantification was performed using the 2^−ΔΔCt^ method.

### 2.4. Statistical Analysis

Normality of data distribution was checked with the Shapiro–Wilk test. For normally distributed variables, ANOVA followed by Tukey’s post hoc test was used; for nonparametric data, Kruskal–Wallis and Dunn’s post hoc tests were applied. Categorical variables were compared with the chi-square test.

Results are expressed as mean ± SD or median (IQR). Diagnostic performance of the analyzed miRNAs was analyzed using receiver operating characteristic (ROC) curves, and area under the curve (AUC) values were interpreted according to standard guidelines. ROC *p*-values were calculated using exact binomial test with 5000 bootstrap resampling iterations. Linear associations among miRNAs were examined using Pearson correlation analysis. Principal component analysis (PCA) was performed on z-score-normalized miRNA expression data to explore overall variance and examine group-specific expression patterns. Adjusted *p*-values were calculated using the Benjamini–Hochberg False Discovery Rate (FDR) method to control type I error due to multiple testing across group comparisons. A *p* value below 0.05 was considered statistically significant. All analyses were performed using Statistica 13.3.1 (StatSoft) and GraphPad Prism 10.0 under the supervision of the investigator.

## 3. Results

### 3.1. Comparable Demographic Profiles

Although age and gender distributions did not differ significantly among the study groups (*p* > 0.05), the results should be interpreted with caution due to sample size variation, particularly in the control group. Nevertheless, the absence of significant demographic differences suggests that observed changes in miRNA expression are more likely related to disease status rather than demographic bias.

### 3.2. Distinct miRNA Expression Profiles Define Disease Groups

**Figure 1:**
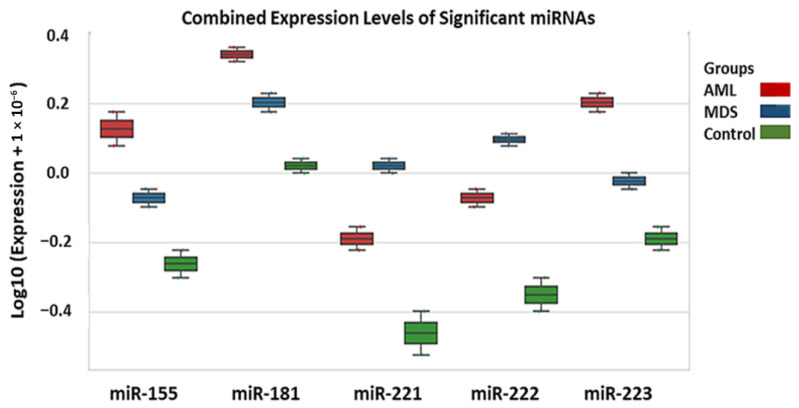
Distribution of log_10_-transformed miRNA expression levels across AML, MDS, and control groups. Box plots represent relative expression of five miRNAs. Log_10_ transformation was applied to normalized 2^−ΔΔCt^ values for visualization purposes. Statistical analyses, however, were performed using untransformed 2^−ΔΔCt^ data; therefore, the numerical mean values reported in Section 3.2 reflect statistical calculations and may not correspond directly to the visual values displayed in the figure.

The expression profiles of miR-155, miR-181, miR-221, miR-222, and miR-223 were compared across AML, MDS, and control groups (Figure 1). miR-155, miR-181, and miR-223 showed gradual increases from control → MDS → AML, whereas miR-221 and miR-222 were markedly reduced in AML compared with both MDS and controls. Among AML samples, miR-155 and miR-223 exhibited higher expression levels, whereas miR-221 and miR-222 were relatively reduced compared with MDS and controls. Numerical comparisons reflect 2^−ΔΔCt^ analysis, while log_10_-transformed values are shown visually in Figure 1 for clarity. This indicates that increased miR-155 and miR-223 together with reduced miR-221/222 define a leukemia-associated transcriptional profile.

### 3.3. Differential Expression and Fold Change Analysis of Selected miRNAs

To identify significantly altered miRNAs, one-way ANOVA followed by Benjamini–Hochberg correction was used (Table 3). All five miRNAs showed significant differences among groups (adjusted *p* < 0.05). Post hoc Tukey HSD analysis (Table 4) revealed the following:

miR-155 was upregulated in AML vs. Control (adj. *p* = 0.012; fold change = 2.45; 95% CI [0.38–0.72]).

miR-181 showed statistically significant differences across all groups (adj. *p* < 0.05; fold change range 1.38–2.10 based on 2^−ΔΔCt^ values), suggesting context-dependent modulation rather than strict directional change.

miR-221 and miR-222 were downregulated in AML compared with Control (adj. *p* = 0.0409 and 0.0195; fold change = 0.65 and 0.62, respectively), but upregulated in MDS vs. Control (adj. *p* = 0.0084 and 0.0051).

miR-223 was downregulated in AML vs. Control (adj. *p* = 0.0046; fold change = 0.43) and showed a modest reduction vs. MDS (adj. *p* = 0.022; fold change = 0.84).

It should be noted that the 95% confidence intervals reported in Table 4 refer to mean differences calculated on the log-transformed scale; therefore, they are not directly comparable to fold change ratios. Fold change interpretation is based on untransformed 2^−ΔΔCt^ values. Statistical significance (*p* < 0.05) was determined using adjusted *p*-values, which remain valid even if confidence intervals include zero on the mean-difference scale.

**Figure 2:**
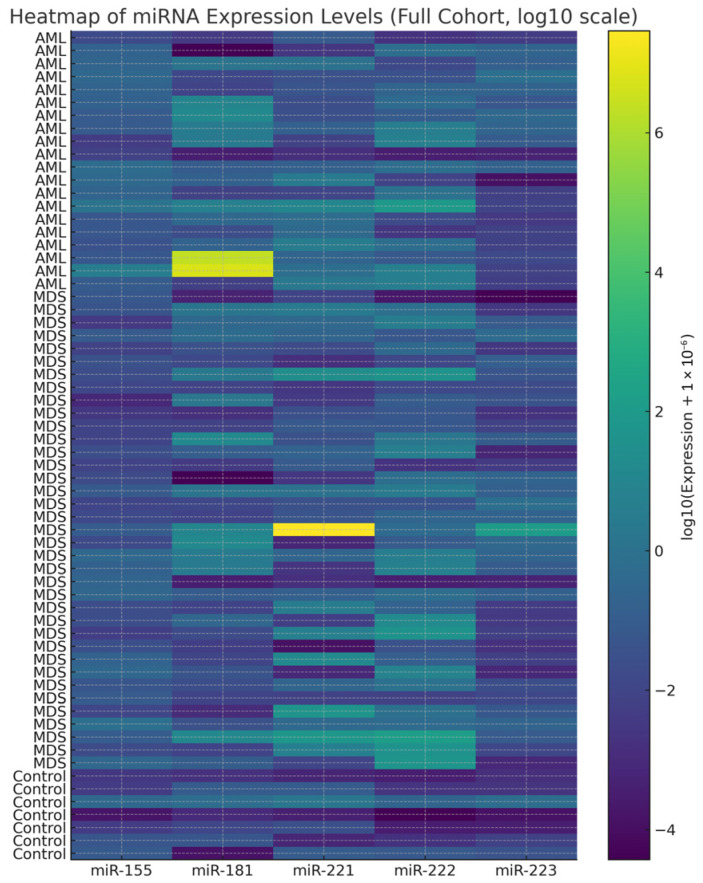
Heatmap of log_10_-transformed 2^−ΔΔCt^ miRNA expression values across all samples in AML (*n* = 20), MDS (*n* = 37), and control (*n* = 7) groups. Each row corresponds to an individual sample and each column to one of the five miRNAs (miR-155, miR-181, miR-221, miR-222, miR-223). Values were log-transformed for visualization purposes only; all statistical analyses were conducted using untransformed 2^−ΔΔCt^ values. Group-specific clustering patterns are observable, with higher expression of miR-155 and miR-223 in AML and reduced expression of miR-221/222 compared with MDS and controls.

**Figure 3:**
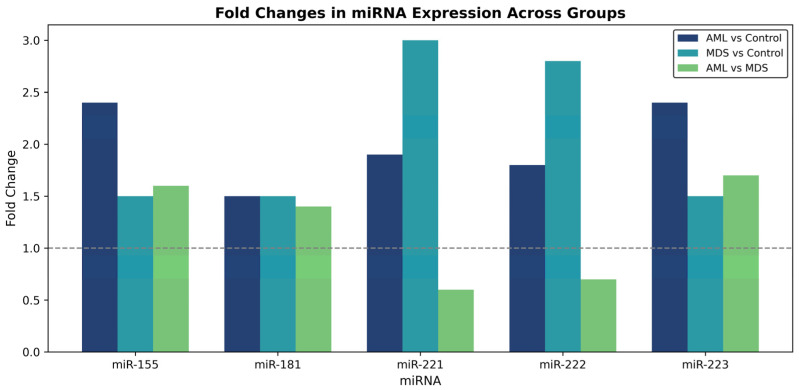
Fold-change comparison of miRNA expression between AML, MDS, and control groups. Bars above and below one indicate upregulation and downregulation, respectively. The dashed line indicates a fold change of 1, representing no change in expression.

Fold change values below 1 indicate downregulation based on the 2^−ΔΔCt^ method. Overall, AML samples were characterized by upregulation of miR-155 and partial elevation of miR-181, together with downregulation of miR-221, miR-222, and miR-223 (Figure 2 and Figure 3). For heatmap visualization, 2^−ΔΔCt^ expression values from all individual patients were used. A log_10_ transformation was applied to improve interpretability (log_10_(expression + ε); ε = 1 × 10^−6^). MDS values were generally intermediate between those of AML and controls, suggesting a gradual shift in miRNA profiles during malignant progression.

### 3.4. Co-Regulatory Relationships Among miRNAs

**Figure 4:**
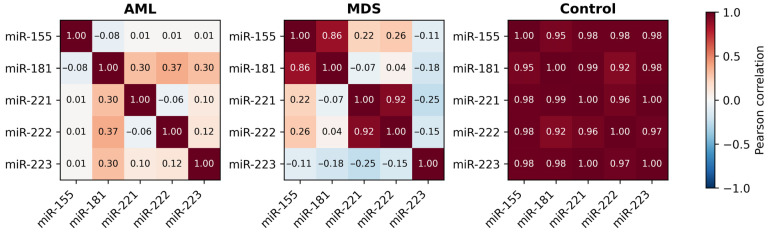
Correlation matrix of miRNA expression in AML, MDS, and control groups. The heatmap illustrates pairwise Pearson correlation coefficients among miR-155, miR-181, miR-221, miR-222, and miR-223 (red: positive; blue: negative). Correlation patterns in the control group should be interpreted cautiously, considering the small sample size.

Pairwise Pearson correlation analysis was performed to determine the relationships between miRNA expression levels (Figure 4). In the control group, all miRNAs were found to be strongly correlated (r > 0.95). This result indicating the presence of a tightly coordinated miRNA network under normal conditions. While weaker coordination was observed in MDS, the strongest correlation was between miR-221 and miR-223. Other miRNA pairs showed only modest correlations. In AML, two distinct positively correlated clusters emerged: miR-155/miR-181 and miR-221/miR-222. miR-223 showed weak or negative correlations with other miRNAs, pointing to a disruption in the regulatory balance. Overall, the data indicate a progressive loss in coordinated miRNA control from normal hematopoiesis to MDS and AML.

### 3.5. Diagnostic Value of miRNAs (ROC Curve Analysis)

**Figure 5:**
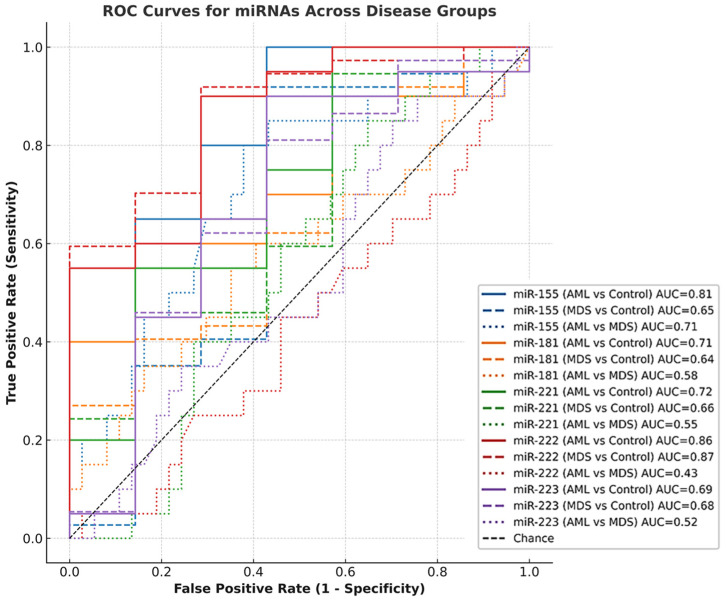
Receiver operating characteristic (ROC) curves for the diagnostic performance of five miRNAs (miR-155, miR-181, miR-221, miR-222, miR-223) across three pairwise comparisons: AML vs. Control (solid line), MDS vs. Control (dashed line), and AML vs. MDS (dotted line). Each miRNA is represented by a distinct color. AUC values are annotated alongside each curve. Analyses were conducted using full-cohort 2^−ΔΔCt^ expression values. Only statistically meaningful patterns are emphasized. The diagonal line represents the reference (AUC = 0.50).

ROC analysis showed that miR-222 exhibited the highest diagnostic accuracy, with AUC values of 0.86 (AML vs. Control; *p* = 0.006) and 0.87 (MDS vs. Control; *p* = 0.002). miR-155 demonstrated moderate but significant discriminatory power (AML vs. Control: AUC = 0.81, *p* = 0.017; AML vs. MDS: AUC = 0.71, *p* = 0.009). In contrast, miR-181, miR-221 and miR-223 showed AUC values between 0.44 and 0.72, all with *p* > 0.05, thus lacking statistical significance. These findings indicate that miR-222 and to a lesser extent miR-155 are the most reliable markers in differentiating disease groups in this cohort (Figure 5; Table 5).

### 3.6. Principal Component Analysis (PCA)

**Figure 6:**
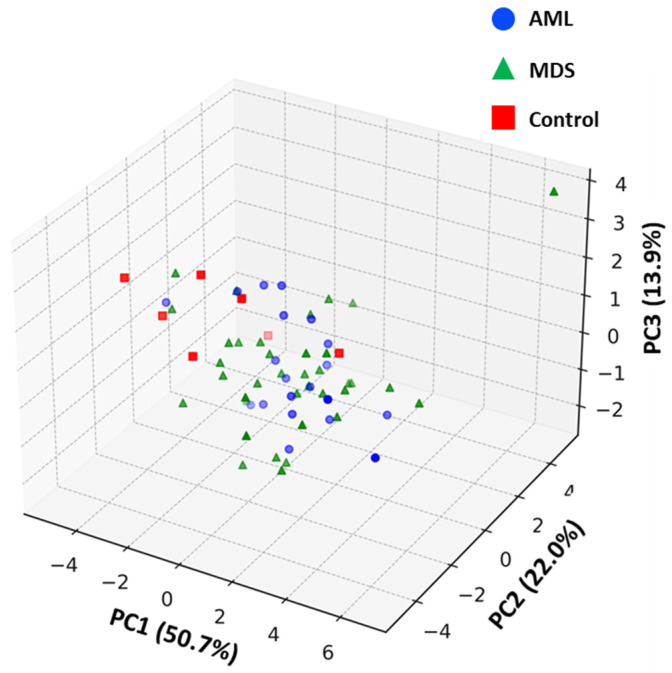
Three-dimensional principal component analysis (PCA) of log_10_-transformed 2^−ΔΔCt^ miRNA expression values across AML (*n* = 20), MDS (*n* = 37), and control (*n* = 7) samples. Axes represent the first three principal components (PC1: 50.7%, PC2: 22.0%, PC3: 13.9% of total variance). Control samples partially overlap with AML along PC1, suggesting expression-level similarity. PCA was performed using eigenvalue > 1 as retention criterion.

Principal component analysis was performed to explore global miRNA expression patterns across the study groups (Figure 6). The first three principal components accounted for 86.6% of the total variance (PC1: 50.7%, PC2: 22.0%, PC3: 13.9%) and were retained based on eigenvalues > 1 and biological interpretability.

AML and MDS samples demonstrated a gradual separation trend along PC1 and PC2, consistent with disease progression patterns. Control samples partially overlapped with AML cases, particularly along PC1, which likely reflects biological proximity in basal hematopoietic expression profiles and is further influenced by the limited control group size (*n* = 7), rather than a visualization artifact.

miR-155 and miR-222 showed the strongest contribution to PCA-based clustering, aligning with their diagnostic relevance observed in ROC and expression analyses. Overall, PCA supported disease-associated shifts in miRNA profiles, although complete group separation was not observed using unsupervised clustering alone.

### 3.7. miRNA Expression and Disease Risk in MDS

In MDS patients, IPSS-R risk groups were compared to determine the clinical relevance of miRNA expression levels (Table 6). miR-222 expression was significantly higher in the high IPSS-R risk categories (adj. *p* = 0.046). A similar trend was observed for miR-221, although the increase was borderline significance (adj. *p* = 0.054). No statistically significant differences were found in the expression levels of the other miRNAs (miR-155, miR-181, and miR-223) across the IPSS risk groups (*p* = 0.245–0.334). These findings suggest that miR-222, and possibly miR-221, are associated with higher disease risk and may serve as molecular indicators of MDS with high IPSS-R scores. This pattern is consistent with the PCA results, which identified miR-222 as a marker reflecting disease progression and heterogeneity.

## 4. Discussion

### 4.1. Diagnostic and Biological Interpretation of miRNA Alterations

In this study, we demonstrated a characteristic expression pattern of five key miRNAs involved in myeloid regulation, characterized by increased miR-155, miR-181 and miR-223 expression, alongside reduced miR-221 and miR-222 levels in AML compared with controls. This pattern is broadly consistent with previous evidence associating miR-155 with leukemic progression and inflammatory amplification through NF-κB/STAT5 signaling [10], as also reinforced by recent data highlighting its relevance in aggressive AML phenotypes [27]. Interestingly, although miR-155 expression showed a clear increase in AML in our cohort, we did not observe significant elevation in high-risk MDS. This may indicate that full leukemic transformation is required for its activation, aligning with findings from Amiri et al. [6], who reported higher miR-155 levels primarily in established AML rather than preleukemic states.

miR-181 was similarly increased in AML and moderately elevated in MDS compared with controls, reflecting its recognized context-dependent effects. While earlier studies described reduced miR-181 expression in high-risk MDS [15], others reported pro-leukemic activity under certain mutational backgrounds [28], suggesting that miR-181 may contribute to disease evolution via threshold-dependent modulation of differentiation regulators such as BCL2 and MEIS1. Recent laboratory data further suggest that miR-181-mediated effects may be accentuated in patients with specific pathway alterations [13,27], which could explain its intermediate profile in our MDS cohort.

The decrease in miR-221 and miR-222 observed in AML is in agreement with functional evidence describing reduced c-KIT–mediated signaling and disruption of cell-cycle regulation upon their downregulation [19,21,29]. Although our study demonstrated relatively higher miR-221/222 expression in MDS compared with AML, consistent with earlier reports suggesting partial preservation of differentiation control in preleukemic states, their decline in IPSS-R high-risk cases may indicate progressive derepression of proliferative pathways during disease evolution. While direct evidence linking these miRNAs to IPSS-R stratification remains limited, risk-associated miRNA upregulation has been demonstrated for other candidates such as miR-196b-5p in high-risk MDS and secondary AML [17]. These findings align with recent high-throughput miRNA profiling studies showing transcriptional drift toward leukemic signatures in advanced dysplasia [9,30], although our cohort did not demonstrate complete convergence to AML profiles.

miR-223 was significantly upregulated in AML, consistent with compensatory regulatory activity in response to dysregulated differentiation pathways [22,23,24]. However, because reduced serum miR-223 has been linked to poor prognosis [25], the observed increase in bone marrow tissue may represent an adaptive response rather than a universally favorable indicator. This interpretation is consistent with reports indicating context-dependent dysregulation of miR-223 across AML subgroups.

Collectively, our integrative evaluation supports the concept that disease progression from MDS to AML is characterized not only by altered expression levels of individual miRNAs but also by progressive disruption of coordinated regulatory networks, as reflected by our correlation analysis and further supported by PCA-based clustering. This interpretation is further supported by the correlation matrix (Figure 4), where tightly coordinated expression observed under normal conditions weakened in MDS and segregated into two distinct clusters in AML (miR-155/miR-181 and miR-221/miR-222), indicating early fragmentation of network stability. Correlation findings in controls should be interpreted with caution due to the small sample size. These findings support previously suggested models of transcriptional instability as an early hallmark of malignant transition [31], reinforcing the need for network-level profiling rather than single-miRNA assessment.

### 4.2. Clinical and Translational Implications

From a diagnostic perspective, the present findings reinforce the potential utility of integrating specific miRNA signatures into established risk stratification systems. In our cohort, miR-155 and miR-222 achieved the highest diagnostic performance (AUC ≥ 0.80) in distinguishing malignant from control bone marrow, confirming their translational relevance. These results are consistent with mechanistic data showing that miR-222 promotes leukemic proliferation via WNT/β-catenin activation [21] and that miR-155 drives NF-κB/STAT5-mediated clonal expansion in AML [10]. Furthermore, accumulating evidence suggests that miRNA profiling may serve as a complementary diagnostic adjunct in hematological settings, particularly when combined with cytogenetic and established scoring tools. In the context of disease progression, miR-222 expression was significantly higher in IPSS-R high-risk MDS, supporting the notion that miRNA reprogramming intensifies with dysplasia severity. Although AML profiles were globally distinct from MDS, IPSS-R high-risk MDS exhibited a partial shift toward AML-like expression trends, suggesting transcriptional priming toward leukemic evolution. This observation is in line with recent multidimensional analyses demonstrating gradual reconfiguration of miRNA regulatory networks during MDS progression [30]. However, the expression pattern was not identical to AML, indicating that while dysplasia may initiate transcriptional reorganization, additional molecular lesions are likely required to enable full leukemic conversion, as supported by recent models of transformation at the stem cell level [32] and integrated multi-omic evidence.

Regarding Table 6, our data indicate that although IPSS-R high-risk MDS miRNA profiles are closer to AML than to low-risk MDS, they differ sufficiently to support the concept that MDS represents a biologically heterogeneous precursor state rather than a uniform malignancy. This is further supported by our correlation and PCA analyses, in which network destabilization appeared earlier than complete expression shifts, suggesting that loss of coordinated miRNA regulation may be an early marker of clonal evolution.

Although detailed genetic stratification was not available due to the timing of patient recruitment, the observed expression trends remained consistent with known molecular transformation patterns. Finally, the translational potential of co-miRNA profiling is reinforced by growing interest in therapeutic modulation of miRNAs in MDS and AML. When integrated with molecular scoring systems such as IPSS-R, composite miRNA signatures may facilitate earlier identification of high-risk transformation and enable personalized treatment planning, supporting their progressive integration into future precision hematology workflows.

### 4.3. Limitations and Future Perspectives

Several limitations of this study should be acknowledged. First, the sample size was modest and derived from a single center, which may restrict generalizability. Due to ethical constraints, the number of healthy bone marrow controls was limited (*n* = 7), which may have contributed to elevated correlation values through reduced biological variability. As patient recruitment was completed prior to 2022, all diagnoses were based on WHO 2016 criteria. While WHO 2022 and ICC 2022 introduced further molecular refinements, genetic data stratifying AML-MR versus de novo AML were not systematically available; therefore, subgroup-based miRNA comparisons should be interpreted within this operational limitation.

Second, although the five-miRNA panel provided meaningful diagnostic and biological insights, additional miRNAs such as miR-29, miR-125b or miR-451 may improve predictive robustness, as demonstrated in recent multi-marker analyses [30]. Moreover, the present results are based solely on expression profiling and lack functional validation, indicating the need for mechanistic follow-up studies. The therapeutic relevance of miRNA modulation has been suggested by experimental observations in AML models, highlighting the potential of miRNA-targeted interventions to influence drug response. Another point to consider is the variability in miRNA expression depending on sampling source and disease kinetics. Several recent studies have emphasized that prognostic performance may differ between bone marrow and peripheral blood [8,25], and between diagnosis and relapse phases, supporting the value of longitudinal monitoring designs.

Lastly, while standard univariate statistical comparisons and ROC analyses were appropriate for this exploratory study, emerging machine learning–based approaches may offer superior discriminatory capability and should be considered in future research, particularly when integrated with genomic and transcriptomic profiling.

Despite these limitations, this work contributes to the expanding literature by providing bone marrow–based evidence of coordinated miRNA dysregulation across the MDS–AML continuum. Future studies should validate these findings in larger, genetically stratified cohorts, include serial sampling, and assess the integration of miRNA profiles with genomic classifiers and emerging therapeutic targets.

## 5. Conclusions

This study demonstrates that dysregulation and loss of coordinated control among miR-155, miR-181, miR-221, miR-222, and miR-223 reflect the gradual transition from MDS to AML. In particular, miR-155 and miR-222 showed the most consistent diagnostic performance, suggesting their value as early indicators of malignant evolution. While expression patterns in high-risk MDS partially shifted toward those seen in AML, complete alignment was not observed, supporting the view that miRNA alterations alone are insufficient for complete leukemic conversion.

Although this study does not propose new mechanistic pathways, it refines current understanding by showing that combined miRNA signatures may complement existing risk models when interpreted alongside clinical data. However, the absence of systematic genetic subclassification (e.g., AML-MR vs. de novo AML) limits further biological interpretation.

In summary, composite miRNA profiling appears promising for early detection and risk refinement in MDS and AML, but confirmation in larger, genetically stratified cohorts is required prior to clinical application.

## Figures and Tables

**Table 1:** Clinical characteristics of patients with MDS and AML.

Parameter	MDS (*n* = 37)	AML (*n* = 20)
Gender (Male/Female)	20/17	10/10
Age (years)	69 (50–88)	63 (43–77)
At age of diagnosis (years)		
≤65	12	7
>65	25	13
WHO 2016/FAB classification		
MDS-RS-SLD	5	M0: 7
MDS-MLD	17	M1: 5
MDS-EB1	12	M4: 5
MDS-EB2	3	M5: 3
IPSS-R/Cytogenetic risk		
Very low	4	Good risk: 3
Low	14	Intermediate risk: 14
Intermediate	15	Poor risk: 3
High & Very high	4	
Number of cytopenias		
0	5	–
1	16	–
2	14	–
3	2	–
Bone marrow blasts (%)		
<5%	22	–
≥5% and <10%	12	–
≥10% and <29%	3	–
Hemoglobin (g/dL)	Low: 29; Normal: 8	Low: 17; Normal: 3
Platelets (×10^3^/L)	≤150 − 17; >150 − 20	≤150: 8; >150: 12
Absolute neutrophil count (×10^3^/L)	≤1.5 − 10; 1.5–6.7 − 21; >6.7 − 6	≤1.5 − 4; 1.5–6.7 − 12; >6.7 − 4

MDS: myelodysplastic syndromes. RS-SLD: ring sideroblasts with single-lineage dysplasia; MLD; multilineage dysplasia; EB: excess blasts; FAB: French–American–British; IPSS-R: Revised International Prognostic Scoring System. AML risk groups were classified according to the ELN 2017 risk stratification system. Low hemoglobin was defined according to WHO anemia criteria (<12 g/dL for females, <13 g/dL for males).

**Table 2:** Primer and probe sequences used for RT-qPCR.

Gene	Primers and Probe Sequences
hsa-miR-155-5p (406947) *	R:5′-TCGTATGCAGTGCAGGGTCCGAGGTATTCGCACTGCATACGAC ACCCCT-3′ F: 5′-GCCGCTTAATGCTAATCGTGAT-3′ PR: 5′-FAM-TG(pdC)ATA(pdC)GA(pdC)A(pdC)C(pdC)(pdC)TAT -BHQ-1-3′
hsa-miR-181a-5p (406954) *	R:5′-GTCGTATGCAGTGCAGGGTCCGAGGTATTCGCACTGCATACGACACTCAC-3′ F:5′-GCCGCAACATTCAACGCTGTCG-3′ PR:5′-FAM-TG(pdC)ATA(pdC)GA(pdC)ACT(pdC)ACCGA-BHQ-1-3′
hsa-miR-221-5p (407006) *	R:5′-TCGTATGCAGTGCAGGGTCCGAGGTATTCGCACTGCATACGACGAAACC-3′ F:5′-GCCGCAGCTACATTGTCTGCTG-3′ PR:5′-FAM-TG(pdC)ATA(pdC)GA(pdC)GAAA(pdC)CCA-BHQ-1-3′
hsa-miR-222-5p (407007) *	R:5′-TCTATGCAGTGCAGGGTCCGAGGTATTCGCACTGCATACGACACCCAG-3′ F:5′-GCCGCAGCTACATCTGGCTA-3′ PR:5′-FAM-TG(pdC)ATA(pdC)GA(pdC)ACC(pdC)AGTAGC-BHQ 1-3′
hsa-miR-223-3p (407008) *	R:5′-TCGTATGCAGTGCAGGGTCCGAGGTATTCGCACTGCATACGACTGGGGT-3′ F:5′-GCCGCTGTCAGTTTGTCAAAT-3′ PR:5′-FAM-TG(pdC)ATA(pdC)GA(pdC)TGGGGTAT-ZNA4-BHQ-1-3′
hsa-miR-26b-5p (407017) *	R:5′-GTCGTATGCAGTGCAGGGTCCGAGGTATTCGCACTGCATACGACACCTAT-3′ F:5′-GCCGCTTCAAGTAATTCAGG-3′ PR:5′-FAM-TG(pdC)ATA(pdC)GA(pdC)A(pdC)CTATCC-ZNA4-BHQ-1-3′

* Gene ID: http://www.ncbi.nlm.nih.gov/gene (accessed on 10 July 2020). R: Reverse primer, F: Forward primer, PR: Probe.

**Table 3:** One-way ANOVA results comparing miRNA expression among AML, MDS, and control groups.

miRNA	F-Statistic	Original *p*-Value	Adjusted *p* Value
miR-155	24.8508	0.0136	0.0136
miR-181	50.0209	0.0050	0.0093
miR-221	32.0273	0.0095	0.0118
miR-222	46.0810	0.0056	0.0093
miR-223	48.6694	0.0052	0.0093

Adjusted *p*-value: Corrected for multiple comparisons using the Benjamini–Hochberg method (FDR control).

**Table 4:** Tukey HSD multiple comparison results and fold-change values for miRNA expression across AML, MDS, and control groups.

miRNA	Comparison	Mean Difference	Adj. *p*	95% Confidence Interval	Significant?	Fold Change
miR-155	AML-Control	−0.3891	0.012	[−0.6197, 0.1584]	Yes	2.45
	AML-MDS	−0.1990	0.0727	[−0.4296, 0.0317]	No	1.59
	Control-MDS	0.1901	0.0813	[−0.0405, 0.4207]	No	1.55
miR-181	AML-Control	−0.3213	0.0044	[−0.4559, 0.1866]	Yes	2.10
	AML-MDS	−0.1387	0.0463	[−0.2733, 0.0041]	Yes	1.38
	Control-MDS	0.1826	0.0221	[0.0479, 0.3172]	Yes	1.52
miR-221	AML-Control	−0.2720	0.0409	[−0.5239, 0.0201]	Yes	1.86
	AML-MDS	0.2091	0.0800	[−0.0428, 0.4610]	No	0.62
	Control-MDS	0.4811	0.0084	[0.2292, 0.7330]	Yes	3.00
miR-222	AML-Control	−0.2782	0.0195	[−0.4743, 0.0820]	Yes	1.89
	AML-MDS	0.1679	0.0741	[−0.0282, 0.3640]	No	0.68
	Control-MDS	0.4460	0.0051	[0.2499, 0.6422]	Yes	2.78
miR-223	AML-Control	−0.3916	0.0046	[−0.5582, 0.2251]	Yes	2.46
	AML-MDS	−0.2261	0.0220	[−0.3927, 0.0596]	Yes	1.68
	Control-MDS	0.1655	0.0508	[−0.0010, 0.3320]	No	1.46

Adj. *p*: Adjusted *p*-value (Benjamini–Hochberg method); CI (95%): 95% confidence interval for mean difference; Significant: “Yes” if *p* < 0.05; Fold Change: 2-based antilog of log_2_ expression difference.

**Table 5:** ROC analysis of diagnostic performance of candidate miRNAs.

miRNA	AML vs. Control (AUC, *p*)	MDS vs. Control (AUC, *p*)	AML vs. MDS (AUC, *p*)
**miR-222**	**0.86 (0.71–0.97) *p* = 0.006**	**0.87 (0.74–0.96) *p* = 0.002**	0.43 (0.28–0.59) ns
**miR-155**	**0.81 (0.64–0.93) *p* = 0.017**	0.65 (0.41–0.81) ns	**0.71 (0.53–0.88) *p* = 0.009**
miR-181	0.69 (0.48–0.85) ns	0.72 (0.51–0.88) ns	0.44 (0.31–0.59) ns
miR-221	0.68 (0.49–0.86) ns	0.72 (0.56–0.91) ns	0.45 (0.29–0.63) ns
miR-223	0.66 (0.43–0.82) ns	0.69 (0.47–0.85) ns	0.47 (0.30–0.63) ns

Data are presented as area under the curve (AUC) with 95% confidence intervals. Only miRNAs with AUC ≥ 0.70 and *p* < 0.05 were considered diagnostically significant (Bold). *p*-values were calculated using exact binomial testing, ns = non-significant (*p* > 0.05).

**Table 6:** Differences and statistical associations of selected miRNA expression levels among IPSS-R risk categories in MDS patients.

miRNA	IPSS-R Trend (Very Low to High/Very High)	*p*	Trend Direction	Interpretation
miR-222	2.04 → 45.45	0.046	↑ in high-risk group	Significant association with IPSS-R; markedly upregulated in high/very high-risk MDS.
miR-221	1.94 → 2.81	0.054	↑ in high-risk group	Borderline significance; similar upward trend to miR-222-5p.
miR-181	1.43 → 0.07	0.334	↓ in high-risk group	Not significant; possible early-stage elevation.
miR-155	0.03 → 0.21	0.218	Slight ↑ in high-risk group	Not significant; mild variation among groups.
miR-223	0.03 → 0.06	0.955	–	No difference across IPSS-R categories.

Arrows indicate trends across increasing IPSS-R risk categories: horizontal arrows (→) denote changes from very low to high/very high risk groups, while vertical arrows indicate direction of expression change (↑, increased; ↓, decreased; –, no consistent trend).

## Data Availability

In accordance with institutional ethics approval and patient confidentiality regulations, individual-level data cannot be shared publicly. Aggregated or anonymized data are available from the corresponding author upon reasonable request and with permission from the Mersin University Clinical Research Ethics Committee.
